# RNA blood levels of osteopontin splice variants are cancer markers

**DOI:** 10.1186/2193-1801-2-110

**Published:** 2013-03-14

**Authors:** Franz Hartung, Georg F Weber

**Affiliations:** University of Cincinnati Academic Health Center, College of Pharmacy, 3225 Eden Avenue, Cincinnati, OH 45267-0004 USA

**Keywords:** Biomarker, Blood analysis, Lung cancer, Breast cancer, Cancer progression

## Abstract

**Purpose:**

Despite a sizeable and continuously growing literature on osteopontin and cancer the molecule has not yet found entry into clinical diagnostics. Our identification of spliced variants that are more specific for cancer than the full-length transcript has opened new possibilities for reaching this goal.

**Methods:**

Here we have developed a real-time RT-PCR blood test and evaluated it in a pilot study of breast, lung, pancreatic, gynecologic, and other cancers, compared to non-cancer controls.

**Results:**

Osteopontin-b was increased in lung cancers and pancreatic cancers. When applying a cutoff of 2 standard deviations above normal, elevation in osteopontin-b transcripts detected over 40% of lung cancers. Osteopontin-c was increased in gynecologic and pancreatic cancers. Elevation in osteopontin-c of 2 standard deviations above the normal mean value also detected a fraction of breast cancers and lung cancers, suggesting heterogeneity within those types of tumors. Specifically, breast carcinomas were associated with significantly higher levels of osteopontin-c mRNA in the blood than carcinomas in situ. In lung cancer patients, the osteopontin-c blood RNA levels had an increasing trend with tumor grade.

**Conclusions:**

Osteopontin-b and -c in the blood are biomarkers for distinct cancers. Our investigations may have bearing on cancer screening and diagnosis.

## Introduction

Reliable blood tests for early detection and monitoring of progression are a holy grail in cancer diagnostics. They require the sufficiently abundant presence of a molecule that is uniquely associated with the presence or the progression of a particular type of cancer or multiple cancers. While a plethora of biomarkers has been described in the literature, few have achieved routine clinical use. Among them, PSA for the early detection as well as treatment monitoring of prostate cancer stands out. CEA is elevated in the blood of patients with colorectal, gastric, pancreatic, lung or breast carcinomas. CEA is mainly used to identify recurrence after surgical resection, because the blood test is not reliable as a screening test for early detection. CA125 is a marker for ovarian or endometrial cancers. However, it can also be elevated by uterine fibroids, endometriosis, pelvic inflammatory disease and cirrhosis, as well as in pregnancy and during menstruation.

While biomarkers for specific types of cancer have value, markers that detect multiple cancers may be more beneficial in diagnostic screening, where it is impractical to have one marker for every possible cancer. As such, gene products of cancer progression often are generated as splice variants that are absent from untransformed tissues. Because the same mechanisms for progression are used by various cancers these molecules may broadly indicate the presence of a cancer in the patient.

Osteopontin has been associated with the progression of numerous types of cancer (Weber et al. 2010; Weber et al. 2011; Weber 2011). Although the molecule has been studied as a marker for malignancy over more than 20 years (Craig et al. 1988; Senger et al. 1989) it has not yet found clinical use as a diagnostic. The full-length form of osteopontin (osteopontin-a) acts as a TH_1_ cytokine that may be secreted by macrophages and T-lymphocytes and is elevated in the blood during immune responses, limiting its value as a cancer marker. We have identified the splice variant osteopontin-c (He et al. 2006) to be selectively present in multiple cancers (Sullivan et al. 2009; Tilli et al. 2011) and to serve as a marker for tumor grade (Mirza et al. 2008). These studies were done by immunohistochemistry or real-time RT-PCR on the cancer tissues. The value of the biomarker may be increased if it can be identified in patient blood and can be correlated to clinical determinants of cancer. Because we had previously developed a real-time RT-PCR protocol to quantify the RNA messages of all three osteopontin splice variants in breast cancer specimens, we adapted it to the analysis of total RNA extracted from whole blood. We tested the hypothesis that aggressive cancers continuously shed cells into the circulation, which are detectable by their unique production of spliced osteopontin mRNA.

## Materials and methods

### Patients

Patient samples were obtained under IRB (Institutional Review Board) protocols 04-01-29-01 and 09-11-09-03 (University of Cincinnati). There were several sources of patient blood. Whole blood was collected from breast cancer patients after obtaining informed consent. The blood was frozen immediately on dry ice. Some blood samples (including pancreatic and gynecologic cancers) were also obtained from CHTN (Collaborative Human Tissue Network). Pelleted nucleated cells from lung cancer patients were provided by the University of Cincinnati tissue procurement service. Blood RNA from breast cancer patients and normal controls was obtained from the Division of Human Genetics at The Ohio State University. Other cancers entailed 1 hepatocellular carcinoma and 2 colorectal adenocarcinomas.

### RNA extraction

RNA was extracted from whole blood with RNazol RT (Molecular Research Center Inc.). The extraction of RNA from nucleated cells was accomplished with TriReagent-RT Blood (Molecular Research Center Inc.). No RNA extraction was required for the Ohio State University samples, as they were provided as RNA samples.

### Real-time RT-PCR

All PCR reactions were performed on an ABI Prism 7000 cycler using SYBR Green detection format. cDNA was made from 1 μg RNA in a 20 μl reaction using the iScript reverse transcription kit (BioRad). 5 μl (for actin 2.5 μl) cDNA was added to each PCR reaction in a total volume of 25 μl using the standard PCR buffer system with optimized concentrations of MgCl_2_. For each experiment a no-template reaction was included as a negative control. cDNA from MDA-MB-435 cells, which express endogenous osteopontin splice variants, served as a reference in every run. The primer combinations were described previously (Mirza et al. 2008). The conditions for PCR were 94°C denaturation for 120 s followed by 40 cycles of: 94°C melting for 15 s, 58°C annealing for 30 s, extension at 72°C for 30 s, followed by 72°C for 2 min and 4°C for 5 min, and a melting curve program (the amplification efficiency was only minimally affected by using the same annealing temperature for all PCR reactions, compared to Mirza et al. (2008) which had optimized the annealing temperatures individually; the amplification efficiency is accounted for in the calculations of relative RNA abundance). Product purity, product size, and absence of primer dimers were confirmed by DNA melting curve analysis and agarose gel electrophoresis. Melt curves yielded a single sharp peak for all template reactions, and a minimal melt peak (resulting from primer dimers) or no melt peaks for the no-template control reactions (Mirza et al. 2008). Relative expression ratios of the target genes were calculated from the cycle threshold and efficiency measurements (Pfaffl 2001).

To assess the sensitivity of the assay, we titrated MDA-MB-435 cells, which express all three forms of osteopontin (He et al. 2006) and served here as the reference cell line, into normal blood. RNA was then extracted for real-time RT-PCR. The relative abundance was calculated according to the method of Pfaffl (2001) using the average of all undiluted MDA-MB-435 real-time RT-PCR results (the positive control and reference in all blood RT-PCR runs) as the reference value.

### Statistics

Each data set was analyzed for equality of variances using the Levene test. The Kolmogorov-Smirnov test was applied to determine which of the data sets had a normal distribution. Since none of the datasets followed a normal distribution, they were analyzed with the Kruskal-Wallis Test. A significant value (p<.05) signifies that the medians of the groups being tested are not equal. The non-parametric test uses medians to test the groups rather than the means because medians are robust estimators that are resistant to outliers. In the cases with the significant results (except for single pairwise results), a non-parametric procedure was performed in SAS, PROC MULTTEST, to analyze the differences between the cancer groups and the control by setting up individual contrasts. This is analogous to independent t-tests, in which one controls for the type I error rate using a method such as bootstrapping. The bootstrap method is a non-parametric approach involving multiple re-sampling of the data. Using this SAS procedure, a reproduction of the Dunnett’s test was set up to determine the relationship between osteopontin splice variant levels and clinical variables (tumor stage, tumor grade, primary tumor size), Spearman’s correlation coefficient and its associated p-value were calculated.

## Results

**Figure 1:**
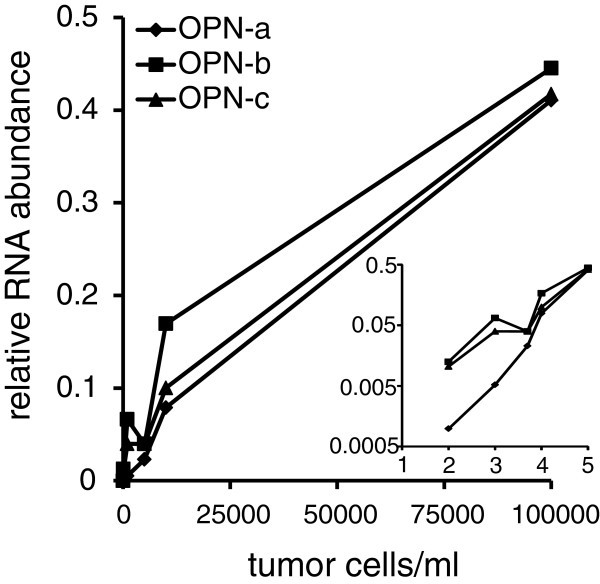
**Assay sensitivity.** Real-time RT-PCR after titration of cancer cells (MDA-MB-435) into blood from a healthy donor. Each data point is the average of three experiments. The PCR reaction was run over 40 cycles, and the reverse transcription reaction contained the standard amount of 1 μg RNA in a 20 μl reaction. The insert shows the data replotted on a double-logarithmic scale. The x-axis labels are 10^×^.

To assess the sensitivity of the method, we collected blood from a healthy donor and titrated increasing numbers of the cancer cell line MDA-MB-435 into it before RNA extraction. This cell line is an abundant producer of all three osteopontin splice variants and therefore allows an estimate of the detection limit of cancer cells in the blood. Because this quantitative PCR method uses MDA-MB-435 cells as its reference, the relative RNA abundance served as a reliable indicator. The actin signal is generated predominantly by the white blood cells and stayed constant regardless of the number of cancer cells added. For each transcript, the CT (cycle threshold) values dropped by about one unit after addition of 100 cancer cells/ml to the blood. However, at the sensitivity of this protocol (applying the algorithm for calculating relative abundance and accounting for assay-to-assay fluctuations) about 1000 cancer cells are reliably detectable by an elevated signal (Figure 1).

**Figure 2:**
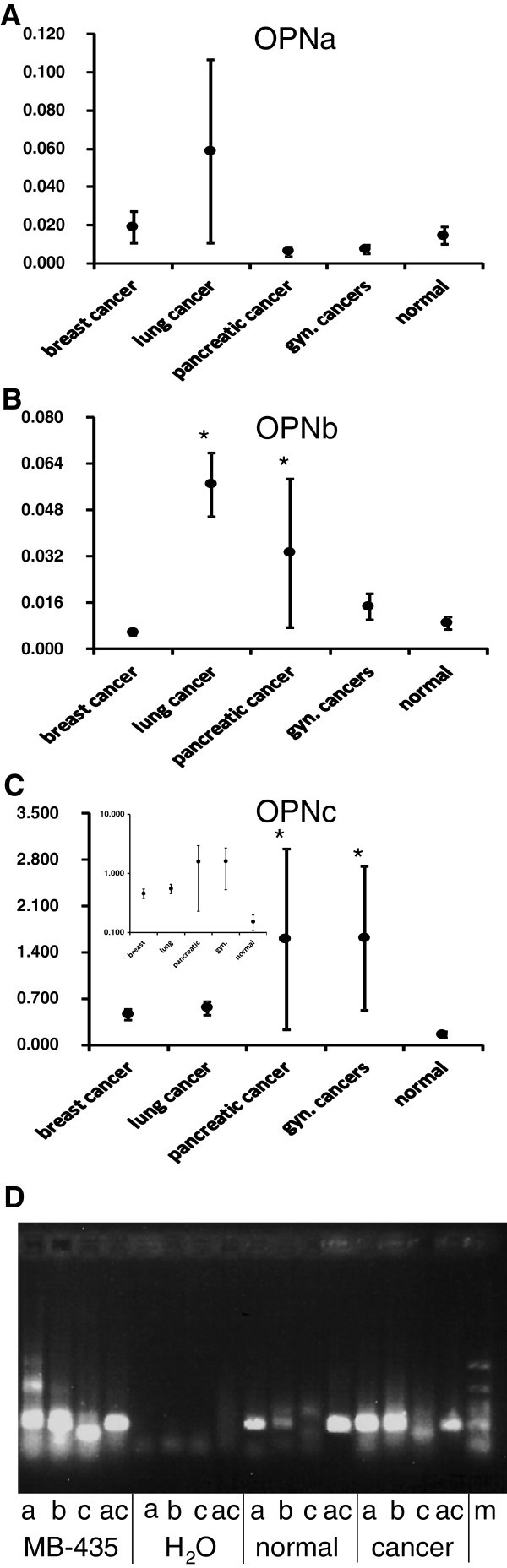
**Osteopontin splice variant RNA in blood.** Analysis of osteopontin splice variant mRNA (mean ± sem) in blood specimens from breast cancer (n = 67), lung cancer (n = 45), pancreatic cancer (n = 7), or gynecologic cancer (n = 6) patients and healthy controls (n = 74). Significance is accepted at the α = 0.05 level. **A**) Measurements of osteopontin-a by real-time RT-PCR in all blood samples. **B**) Osteopontin-b in various cancers versus healthy controls. **C**) Osteopontin-c in various cancers versus healthy controls. The insert shows the same graph with a logarithmic y-axis. **D**) Visualization of representative real-time RT-PCR products by agarose gel electrophoresis. To confirm the integrity of the PCR products, a representative cancer specimen was compared to a representative healthy control. The cell line MDA-MB-435 and no template (H_2_O) served as positive and negative controls respectively. a = ostepontin-a, b = ostepontin-b, c = ostepontin-c, ac = β-actin, m = markers.

The patient demographics are shown in Table 1. We first compared the mRNA abundance for osteopontin splice variants in cancer (breast, lung, pancreatic, gynecologic, other) versus normal (Figure 2). For osteopontin-a, none of the cancers was significantly different from the controls. The large increase of osteopontin-a in the mean value and error bar for lung cancers is caused by an exceedingly high value in one patient, otherwise there was no difference from healthy controls. When considering osteopontin-b, lung cancers, pancreatic cancers, and other cancers were significantly different from controls. Osteopontin-c displayed significant increases from the control group in gynecologic (ovarian, cervical, endometrial) and pancreatic cancers. Although the mean values for osteopontin-c in breast and lung cancers were elevated 3- to 3.5-fold over healthy controls, they did not reach significance. To determine the value of osteopontin splice variants as cancer screening markers, we pooled all cancers and tested for significant differences according to the Kruskal-Wallis test. Whereas osteopontin-a was not significantly different between cancers and controls, osteopontin-b (p= 0.0005) and osteopontin-c (p < 0.0001) were significantly elevated in the cancers.

**Table 1:** **Patient demographics**

		Sex		Age			Race			
	N	Female	Male	Mean	Min	Max	White	Asian	Black	Unknown
**breast cancer**	67	67	0	54.7	24	86	56	2	3	6
**lung cancer**	45	27	18	61.3	35	82	31	0	8	6
**pancreatic cancer**	7	4	3	65.3	43	85	5	0	0	2
**gynecologic cancers**	6	6	0	58.2	39	78	4	0	1	1
**other cancers**	3	0	3	65	53	78	3	0	0	0
**healthy controls**	74	62	7	51.1	18	85	66	2	2	4

For each group, the distribution of sex, age, and race is shown (five of the healthy controls did not have information on sex and age).

Most of the control samples cluster tightly for all three splice variants. The markers have high specificity. Yet, when broadly comparing cancers to controls, the sensitivity is low with only a limited portion of cancer patients showing elevation of one or several osteopontin splice variants in the blood. For the two large groups of breast cancers and lung cancers, we calculated the fraction of patients whose osteopontin RNA message levels were elevated by one, two, or three standard deviations above the mean value of the healthy controls (Table 2). While osteopontin-b and -c capture a fraction of lung cancer patients, osteopontin-c captures a fraction of breast cancer patients. This indicates a heterogeneity within these two groups of cancers that causes osteopontin splice variants to detect subsets within them. Of note, elevation in osteopontin-b RNA is associated with a large fraction of lung cancers, with 56% increased by more than one, 42% more than two, and 24% more than three standard deviations above the mean value of healthy controls.

**Table 2:** **Detection cutoffs**

breast cancer			
	**OPNa**	**OPNb**	**OPNc**
** 1 std**	4% (3)	3% (2)	28% (19)
** 2 std**	3% (2)	1% (1)	15% (10)
** 3 std**	3% (2)	0% (0)	13% (9)
** lung cancer**			
	**OPNa**	**OPNb**	**OPNc**
** 1 std**	7% (3)	56% (26)	38% (17)
** 2 std**	4% (2)	42% (19)	16% (7)
** 3 std**	2% (1)	24% (11)	7% (3)

Percentage (and in parentheses number) of breast cancer or lung cancer patients above a threshold of 1, 2 or 3 standard deviations above the mean value of healthy controls.

We asked whether there are differences among the histologic subtypes of lung cancer with regard to the abundance of osteopontin splice variant RNA in the blood. There were no differences for osteopontin-a, osteopontin-b or osteopontin-c among adenocarcinoma (n = 26), non-small cell lung cancer (n = 4), and squamous cell carcinoma (n = 11) according to the Kruskal-Wallis test (four lung cancers were diagnosed as carcinoid tumor, small cell cancer, pleomorphic carcinoma, non-mucinous bronchioloalveolar carcinoma, and were not included in this analysis). Similarly, there were no differences in abundance of the splice variants between ductal (n = 58) and lobular (n = 7) breast cancers (two cancers were mucinous carcinomas).

**Figure 3:**
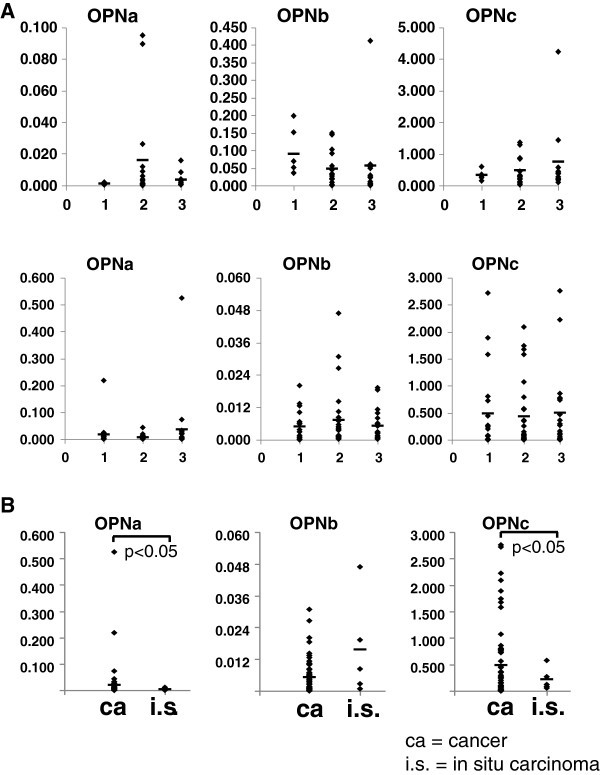
**Osteopontin splice variant RNA in tumor progression. A**) Osteopontin splice variant levels and tumor grade in lung cancers (top panel) and breast cancers (bottom panel). **B**) The levels of osteopontin-a, osteopontin-b, and osteopontin-c in blood from patients with in situ carcinomas and breast cancers. Diamonds are individual data points, horizontal lines represent the mean values (the in situ mean values are ostepontin-a = 0.004, ostepontin-b = 0.016, ostepontin-c = 0.216).

In lung cancers, osteopontin-c increased with higher tumor grades (mean value grade 1 = 0.336, grade 2 = 0.474, grade 3 = 0.743), however, this fell short of reaching statistical significance (Figure 3A). There was no correlation between the levels of osteopontin splice variants and either primary tumor size or tumor stage (Table 3A). In breast cancers, there was no correlation between the levels of osteopontin splice variants and either primary tumor size or tumor stage T or tumor stage N (Table 3B). There was also no correlation with tumor grade (Figure 3A). In situ carcinomas are early forms of breast cancer, which are characterized by the absence of tumor cell invasion into the surrounding tissue. The levels of osteopontin-a and osteopontin-c, but not osteopontin-b, increased from in situ carcinomas to breast cancers (Figure 3B). However, osteopontin-a again showed wide variations with only two patients having dramatically elevated levels. Initially, the paucity of a correlation between osteopontin splice variant expression and tumor progression may be surprising because more cells might be expected to enter the circulation after tumor growth or tumor spread. However, it has been reported that cancers, and even non-transforming diseases, can shed cells into the circulation at early stages (Hardingham et al. 2000; Beitsch and Clifford 2000), suggesting that the burden of circulating cells need not correlate with tumor size or stage.

**Table 3:** **Cancer stage and primary tumor size**

**A. lung cancer**				
**lung cancer stage**		**OPNa**	**OPNb**	**OPNc**
	**correlation coefficient**	0.127	0.172	0.185
	**p-value**	0.480	0.339	0.304
**lung cancer size**		**OPNa**	**OPNb**	**OPNc**
	**correlation coefficient**	-0.193	-0.508	-0.012
	**p-value**	0.259	0.002	0.942
**B. breast cancer**				
**breast cancer stage T**		**OPNa**	**OPNb**	**OPNc**
	**correlation coefficient**	0.223	0.123	0.087
	**p-value**	0.084	0.343	0.507
**breast cancer stage N**		**OPNa**	**OPNb**	**OPNc**
	**correlation coefficient**	0.078	-0.084	0.161
	**p-value**	0.561	0.530	0.227
**breast cancer size**		**OPNa**	**OPNb**	**OPNc**
	**correlation coefficient**	-0.070	-0.090	0.111
	**p-value**	0.585	0.480	0.385

To determine the relationship between osteopontin splice variant levels and tumor stage or primary tumor size, Spearman’s correlation coefficient and its associated p-value were calculated. For lung cancers (A), stage I-IV was used (n = 10, 6, 11, 6 for I, II, II, IV). For breast cancers (B), stage T (n = 37, 16, 8, 2 for T1, T2, T3, T4) and N (n = 28, 20, 5, 7, 0 for N0, N1, N2, N3, N4) were applied to the analysis. Of note, the significant p-value is not reflective of a strong correlation. It indicates that the population correlation coefficient is different than zero with the degree of confidence given by the p-value.

Information on conventional biomarkers was available for several tumors. In the lung cancers, there was no difference in osteopontin-a, osteopontin-b or osteopontin-c between the TFF1+ (n = 12) and the TFF1- (n = 10) subgroups or between the P63+ (n = 8) and P63- (n = 5) subgroups. There were also no differences in osteopontin splice variant levels between CK-7+ (n = 12) and CK-7- (n = 3) lung cancers or between CK-5/6+ (n = 2) and CK-5/6- (n = 5) lung cancers. ER, PR, and HER2 are standard biomarkers for breast cancer that determine treatment decisions. Again, osteopontin-a displayed a wide scatter with outlier samples in the marker-positive and marker-negative groups. In this analysis, osteopontin-c also had one outlier with a high value in the ER- PR- breast cancer group and no significant differences between the groups (not shown). The only significant difference detected was for osteopontin-b between ER- (mean value 0.0098, n = 13) and ER+ (mean value 0.004, n = 49) breast cancers, the relevance of which remains to be elucidated as osteopontin-b was not found to be elevated in breast cancers over control specimens.

## Discussion

Here we describe the real-time RT-PCR detection of osteopontin splice variants in whole blood. While osteopontin-b is a marker for lung cancers and pancreatic cancers. osteopontin-c is increased in gynecologic and pancreatic cancers. However, osteopontin-c also detects fractions of breast cancers and of lung cancers, suggesting heterogeneity within those types of tumors.

Possibly the most striking and unexpected finding of this study was the elevation of osteopontin-b in the blood of a large fraction of lung cancer patients (over 55% elevated more than one standard deviation above the healthy mean value). Osteopontin-b RNA in the blood is a biomarker for lung cancers. Although osteopontin-c was not identified as a marker when considering all lung cancers together, close to 40% of patients had elevated levels of at least one standard deviation above the healthy mean value, and there was a trend toward higher osteopontin-c levels in higher grade cancers. By ELISA (which does not distinguish among splice variants), elevated circulating levels of osteopontin protein have been found to be associated with lung cancer (Chang et al. 2007; Mountzios et al. 2007; Fedarko et al. 2001) and to be predictors of prognosis (Mack et al. 2008). Of note, the osteopontin protein levels in the blood are likely mostly generated by the primary cancer or its established metastases, whereas the blood RNA levels are thought to come entirely from circulating tumor cells.

In breast cancer tissue, osteopontin-c (measured by real-time RT-PCR or immunohistochemistry) is present in over 75% of cases and serves as a marker of tumor grade (Mirza et al. 2008). Unexpectedly, the blood real-time RT-PCR measurements of this study did not find the same correlations. Only about 30% of breast cancers generated blood levels of osteopontin-c that exceeded one standard deviation above the mean value of healthy controls. Clearly, breast cancers were associated with higher osteopontin-c blood RNA levels than in situ carcinomas. Osteopontin-c showed no association with ER, PR, or HER2. While this is in line with its previously reported association with triple negative breast cancer (Mirza et al. 2008; Weber 2011; Weber et al. 2011), there were only five triple negative breast cancers in this study - too few to confirm any association with this subset. Further investigations are required to elucidate the subfractions of breast cancers that cause elevated blood mRNA for osteopontin-c. We have previously found osteopontin-b in breast tissue not to be a marker for breast cancer (Mirza et al. 2008). Consistently, the mean value of osteopontin-b in all breast cancers is not elevated over the mean value in healthy controls, and osteopontin-b does not increase in cancer over in situ carcinomas. Therefore, the difference in osteopontin-b levels between ER+ and ER- breast cancers is surprising. As neither subgroup has significantly higher osteopontin-b values than healthy controls, the difference is not of diagnostic value for cancer.

Our protocol was intended to provide a sensitive screening test that may detect the presence of cancer (without selectivity for a particular type of cancer). While the approach has high specificity, with the available technology it has limited sensitivity. Rather than standing alone as diagnostics in screening, osteopontin splice mRNA blood levels may find use in monitoring cancer progression or in multi-marker panels (see Weber 2011).

For diagnostic applications, it is important that a clinical test be robust to differences in the initial processing of the blood. The material analyzed here came from multiple sources. With the exception of the nucleated cell preparations, all sources of blood RNA had essentially identical mean values and standard deviations for their osteopontin-a and -b real-time PCR results. The RNA extracted from nucleated cells yielded higher values for osteopontin-a and osteopontin-b than the RNA from other sources. We believe this to reflect the high proportion of lung cancers in this sample set rather than a bias in methodology. The samples obtained from CHTN had higher ostoepontin-c measurements than the other sources. Again, that is likely reflective of the cancer selection (all the non-lung, non-breast samples came from that source). CHTN was the only provider that did not guarantee a short time window from blood drawing to freezing. Initially, degradation of osteopontin RNA was a substantial concern. The high values imply that RNA may be more stable after phlebotomy than is generally assumed. Blood RNA, while stable in situ, is believed to degrade rapidly once the blood is drawn. Although we could not detect apparent differences among the samples from various sources, there was a clear loss of osteopontin RNA species (but not of β-actin) when cDNA was thawed for the third time. This loss amounted to an increase in the CT value of 5–10 cycles. Hence, there is limited stability of the osteopontin splice cDNA, which may lead to underestimation if samples are handled improperly. Over 18 runs, the cycle threshold readings for the reference cell line MDA-MB-435 were stable for actin (13.2-14.6), osteopontin-a (13.2-16.7), osteopontin-b (13.6-15.8), and osteopontin-c (13.3-17.1). The data were analyzed by calculating a relative ratio R in various different ways. The method described by Pfaffl (2001) was superior. It uses the amplification efficiencies E and the cycle thresholds CT in the formula

A flexible threshold (set as the inversion point on the logarithmic plot of the amplification curves) did not improve the readings over a fixed threshold. A conventional comparative threshold method is similar to the one used here, but neglects to account for differences in amplification efficiencies by setting E = 2.

## Conclusion

Osteopontin-b and -c in the blood are biomarkers for distinct cancers. Osteopontin-b is increased in lung cancer patients and pancreatic cancer patients. Osteopontin-c is increased in gynecologic and pancreatic cancers, as well as a fraction of breast cancers and lung cancers. Specifically, breast carcinomas are associated with significantly higher levels of osteopontin-c mRNA in the blood than carcinomas in situ. In lung cancer patients, the osteopontin-c blood RNA levels have an increasing trend with tumor grade. Our investigations may have bearing on cancer screening and diagnosis.
